# A new intermediate in the Prins reaction

**DOI:** 10.3762/bjoc.9.51

**Published:** 2013-03-05

**Authors:** Shinichi Yamabe, Takeshi Fukuda, Shoko Yamazaki

**Affiliations:** 1 Fukui Institute for Fundamental Chemistry, Kyoto University Takano-Nishihiraki-cho 34-4, Sakyou-ku, Kyoto 606-8103, Japan; 2 Department of Chemistry, Nara University of Education, Takabatake-cho, Nara 630-8528, Japan

**Keywords:** DFT calculations, hemiacetal intermediate, hydrogen bond, Prins reaction, transition state

## Abstract

Two Prins reactions were investigated by the use of DFT calculations. A model composed of R–CH=CH_2_ + H_3_O^+^(H_2_O)_13_ + (H_2_C=O)_2_, R = Me and Ph, was adopted to trace reaction paths. For both alkenes, the concerted path forming 1,3-diols was obtained as the rate determining step (TS1). TS stands for a transition state. From the 1,3-diol, a bimolecular elimination (TS2) leads to the allylic alcohol as the first channel. In the second channel, the 1,3-diol was converted via TS3 into an unprecedented hemiacetal intermediate, HO–CH_2_–O–CH(R)–CH_2_–CH_2_–OH. This intermediate undergoes ring closure (TS4), affording the 1,3-dioxane product. The intermediate is of almost the same stability as the product, and two species were suggested to be in a state of equilibrium. While the geometry of TS1 appears to be forwarded to that of a carbocation intermediate, the cation disappeared through the enlargement of the water cluster. Dynamical calculations of a classical trajectory using the atom-centered density matrix propagation molecular dynamics model on the four TSs were carried out, and results of IRC calculations were confirmed by them.

## Introduction

**Scheme 1:**
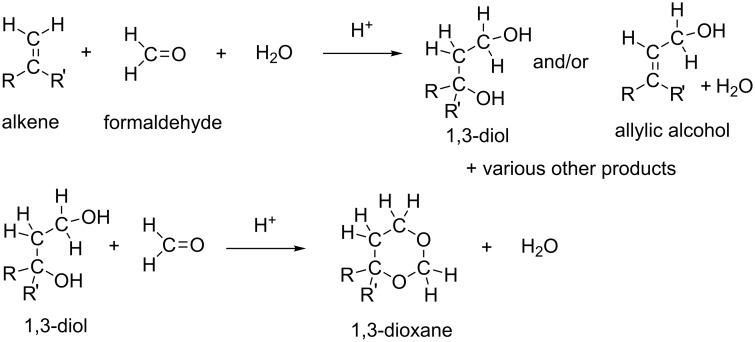
A general scheme of the Prins reaction.

The Prins reaction is the acid-catalyzed addition of aldehydes to alkenes and gives different products depending on the reaction conditions. The first work on the condensation of alkenes with aldehydes was made by Kriewitz in 1899 [1]. He found that unsaturated alcohols were produced when pinene (a bicyclic monoterpene) was heated with paraformaldehyde. However, Prins performed the first rather comprehensive study of the reactions between formaldehyde and hydrocarbons with C=C double bonds [2–3]. These were styrene, pinene, camphene and anethole. As a catalyst, sulfuric acid was used, and water or glacial acetic acid was the solvent. A general Prins reaction is shown in Scheme 1.

**Scheme 2:**
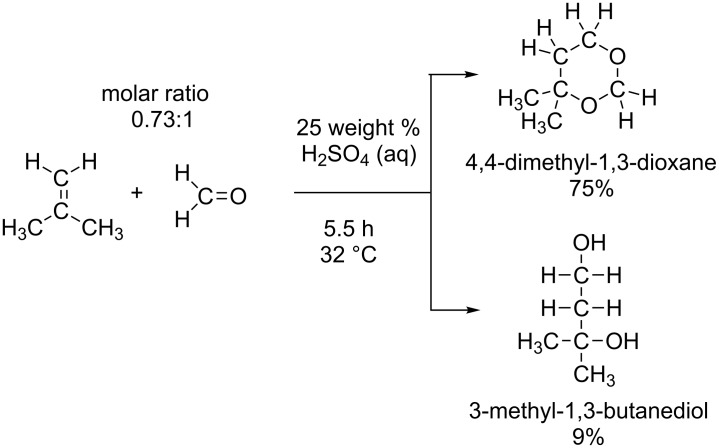
An example of the Prins reaction [4]. The product yields (%) are based on formaldehyde.

**Scheme 3:**
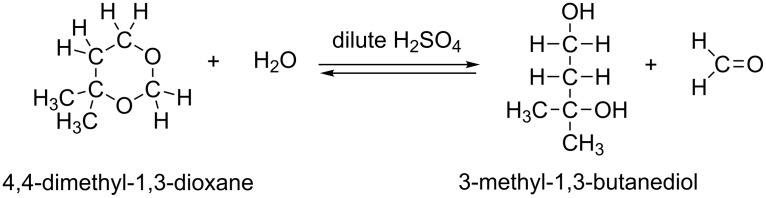
An equilibrium in the hydrolysis of the product, 1,3-dioxane.

A typical Prins reaction is exhibited in Scheme 2 [4]. Here, the six-membered ring compound, 4,4-dimethyl-1,3-dioxane, is the major product along with 3-methyl-1,3-butane-diol. The 1,3-dioxane is hydrolyzed to form the 1,3-diol by stirring the former in a 2% (or lower) sulfuric acid solution under reflux [4]. However, the hydrolysis yield on the dioxane charged is only 8–17% depending on the alkene reactants. The result was interpreted in terms of a reversible reaction shown in Scheme 3.

**Scheme 4:**
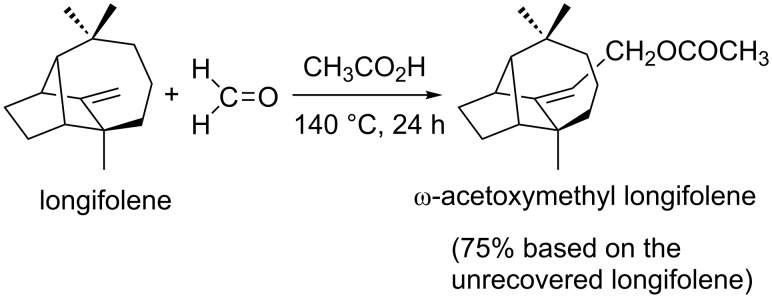
Formation of the acetate of an allylic alcohol by Prins reaction [5].

On the other hand, a reaction of longifolene with formaldehyde in acetic acid yielded the acetate of an allylic alcohol (ω-acetoxymethyl longifolene) as a major product under high-temperature conditions (Scheme 4, 140 °C, 24 h) [5].

**Scheme 5:**
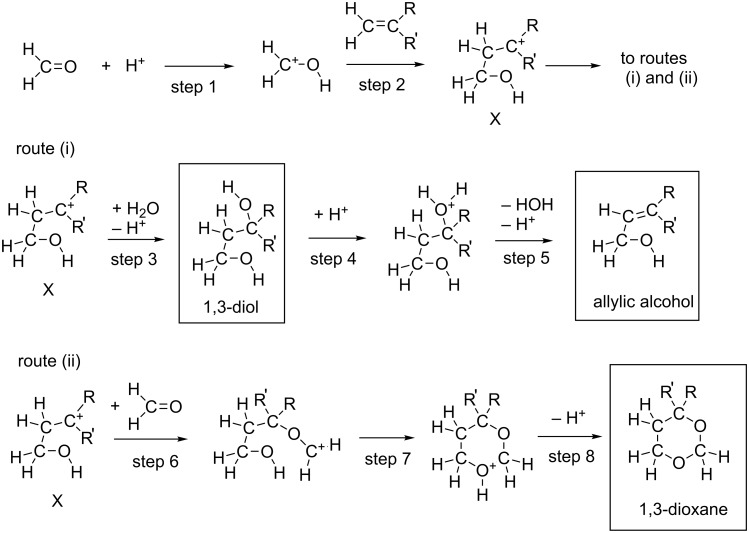
A reaction mechanism involving the carbonium-ion intermediate X.

A scheme was proposed as to the mechanism of the Prins reactions [6–18] to afford 1,3-dioxane, 1,3-diol and allylic alcohol, where the carbocation intermediate (X) is included (Scheme 5).

In the second step, X is formed. From X, two routes, (i) and (ii), are possible. In the route (i), a water molecule is linked with the cation center, which leads to formation of the 1,3-diol and the subsequent allylic alcohol. In (ii), the second H_2_C=O is bound to the cation center, leading to formation of the 1,3-dioxane. While tertiary carbocations might intervene, the presence or absence of secondary ones would be critical in the aqueous media. This is because the water cluster has high nucleophilic strength and tends to make C–O bonds to form alcohols , overcoming the intervention of carbocations. In this respect, the mechanism depicted in Scheme 5 needs to be examined by the use of some alkenes theoretically.

A variety of protic acids and Lewis acids are employed to catalyze the reaction, and Prins-type reactions have found numerous synthetic applications [6,19–25]. In spite of the extensive experimental studies, there have been no computational studies of the classic Prins reaction. The reaction is also important, because the constituent atoms are only C, H and O in the original system, and it is a fundamental organic reaction. In this work, as the first attempt, reactions paths were traced by DFT calculations under Prins’ original conditions (styrene and formaldehyde in the acidic aqueous media). As an alkene, propene was also employed for comparison. From styrene, 4-phenyl-1,3-dioxane was obtained in 86% yield, and from propene 4-methyl-1,3-dioxane in 65% yield (based on H_2_C=O) [4]. The target of this work is to check whether the seemingly established mechanism shown in Scheme 5 holds for the two alkenes and the expected steps in Scheme 5 are obtained by DFT calculations.

## Method of calculations

The reacting systems were investigated by density functional theory calculations. The B3LYP method [26–27] was used for geometry optimizations. In order to check the reliability of B3LYP, M06-2X [28] and a dispersion correction method (ωB97XD [29]) were applied to the rate-determining step of the propene reaction TS1(Me). The basis sets employed were 6-31G(d) and 6-311+G(d,p). Transition states (TSs) were sought first by partial optimizations at bond interchange regions. Second, by the use of Hessian matrices TS geometries were optimized. They were characterized by vibrational analysis, which checked whether the obtained geometries have single imaginary frequencies (ν^‡^s). From the TSs, reaction paths were traced by the intrinsic reaction coordinate (IRC) method [30–31] to obtain the energy-minimum geometries. Relative energies Δ*E* were obtained by single-point calculations of RB3LYP/6-311+G(d,p) [self-consistent reaction field (SCRF) = PCM [32–34], solvent = water] on the RB3LYP/6-31G(d) and 6-311+G(d,p) geometries and their ZPE ones. Here, ZPE denotes the zero-point vibrational energy.

In order to confirm the obtained TS characters, dynamical calculations of a classical trajectory calculation using the atom-centered density matrix propagation molecular dynamics (ADMP) model [35–37] on TSs were carried out. Geometries of the TSs at 2000 steps of 0.1 femtoseconds (10^−15^ seconds) were determined.

All the calculations were carried out by using the GAUSSIAN 09 [38] program package. The computations were performed at the Research Center for Computational Science, Okazaki, Japan.

**Scheme 6:**
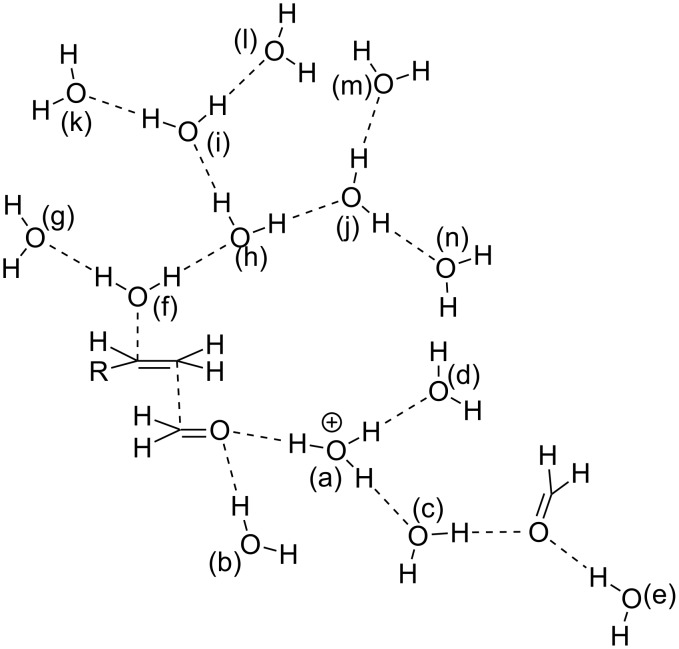
A reaction model composed of RHC=CH_2_, (H_2_C=O)_2_ and H_3_O^+^(H_2_O)_13_ to obtain the path of step 2 (Scheme 5). H_3_O^+^ and H_2_O are labeled with (a), (b), (c) … (n) to explain their positions.

As for the model, alkene, two H_2_C=O and H_3_O^+^ molecules are needed to simulate the paths depicted in Scheme 5. In addition to them, 13 H_2_O molecules are included as shown in Scheme 6. In the model, H_2_C=O catalyzed by H_3_O^+^ (a) works as an electrophile to add to the alkene. The addition follows Markownikoff's rule [39]. H_2_O (f) is the nucleophile to the left-hand carbon of the alkene. One proton of H_2_O (f) moves to H_2_O (h) upon the addition, and this becomes a hydronium ion. Around the newly formed ion, six H_2_O molecules (i, j, k, l, m, and n) are located. At the same time, toward the carbonyl oxygen of the central H_2_C=O, a proton is moved from H_3_O^+^ (a). To this ion, H_2_O (c) and H_2_O (d) are attached. H_2_O (b) and H_2_O (e) are coordinated to the second sp^2^ lone-pair orbitals of two H_2_C=O molecules.

## Results and Discussion

### The propene reaction

**Figure 1:**
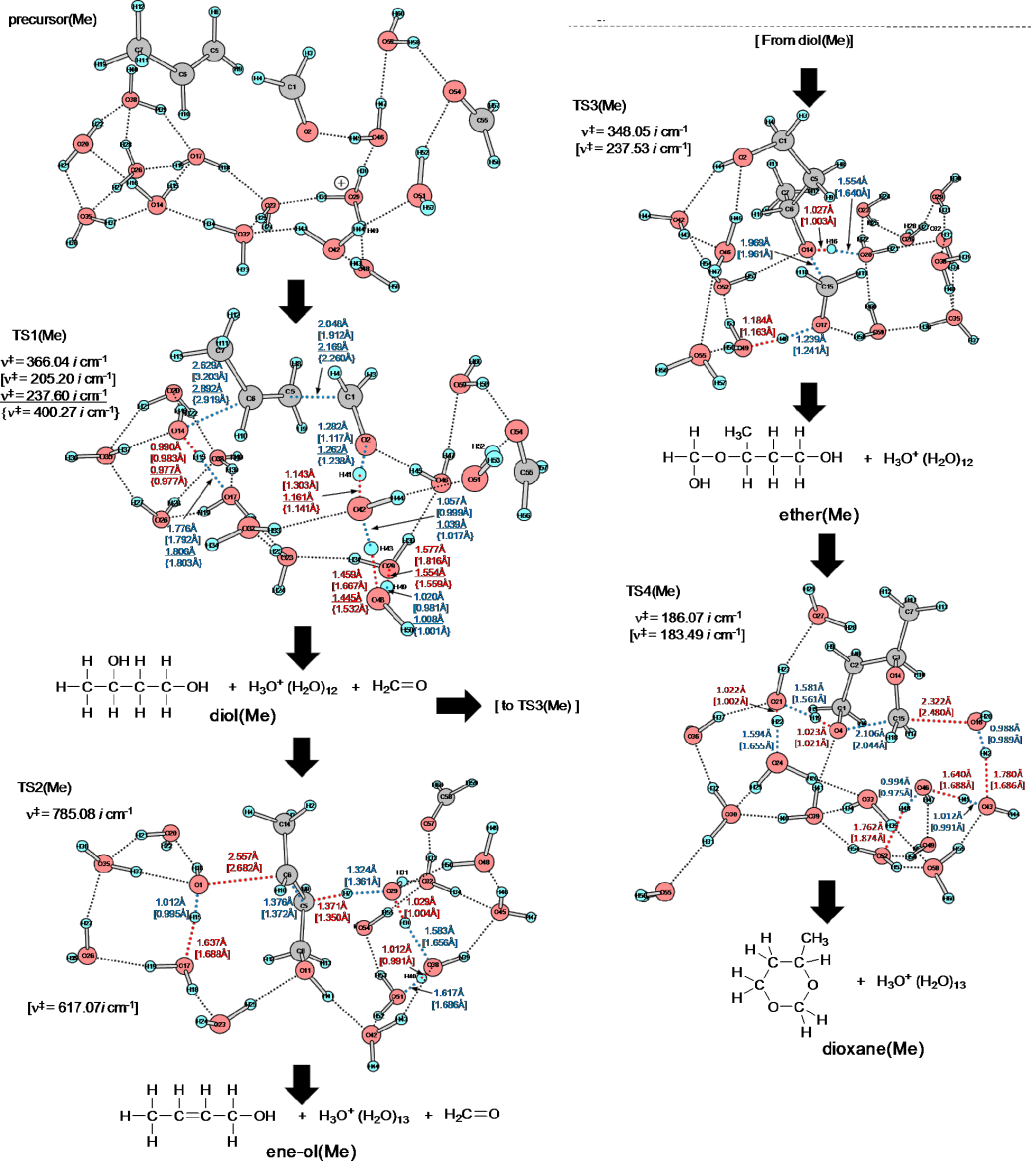
Geometries of the precursor and the transition states (TSs) of the Prins reaction of propene with (formaldehyde)_2_ and H_3_O^+^(H_2_O)_13_ according to the model of Scheme 6. Sole imaginary frequencies, ν^‡^s, verifying that the obtained geometries are at saddle points, are also shown. Those of intermediates and products are exhibited in Figure S1 of Supporting Information File 1. "(Me)" denotes the propene (R–HC=CH_2_, R = Me) reaction. Distances and imaginary frequencies by B3LYP/6-31G(d) and B3LYP/6-311+G(d,p) (in square brackets) are shown. Underlined numbers are by M06-2X and those in braces by ωB97XD for TS1(Me).

Figure 1 shows precursor and TS geometries in a Prins reaction of propene. In precursor (Me), while two H_2_C=O molecules are linked with water ones via hydrogen bonds, hydrophobic propene is outside them. When it is put into the water cluster, the first transition state [TS1(Me)] is brought about. Worthy of note is that various concomitant bond interchanges are involved in TS1(Me). The reaction center is at the C(1)···C(5) bond, and simultaneously the incipient C(6)···O(14) bond is formed. After TS1(Me), not the carbonium ion but rather the butane-1,3-diol, diol(Me), is afforded. This result demonstrates that steps 1, 2 and 3 in Scheme 5 occur at the same time without intervention of the carbocation X. From diol(Me), two TSs were obtained. One is TS2(Me) leading to the allylic alcohol, 2-buten-1-ol, here called ene-ol(Me). The other is TS3(Me) leading to an intermediate, not included in Scheme 5. This species, 3-(hydroxymethoxy)-1-butanol, called here ether(Me), has an ether moiety and is a hemiacetal. Generally, these are formed by the formal addition of an alcohol to the carbonyl group. In this case, 1,3-diol(Me) is the alcohol, and the second formaldehyde is of the C=O group. Closure of the six-membered ring from the ether(Me) [TS4(Me)] gives the product 4-methyl-1,3-dioxane, dioxane(Me). Thus, the obtained route (ii), precursor (Me) → diol(Me) → ether(Me) → dioxane(Me), is different from that in Scheme 5. The first difference is the absence of the carbocation X in the former route. The second one is a new hemiacetal intermediate, ether(Me).

Geometries in Figure S1 (Supporting Information File 1) were obtained by IRC calculations starting from TS ones in Figure 1. In order to confirm the route depicted in Figure 1, ADMP dynamical calculations from TSs were also performed. Geometries after 200 femtosecond from TS1(Me), TS2(Me), TS3(Me) and TS4(Me) are shown as ADMP1(Me), ADMP2(Me), ADMP3(Me) and ADMP4(Me), respectively, in Figure S2 (Supporting Information File 1). These were found to be similar to diol(Me), ene-ol(Me), ether(Me) and dioxane(Me) in Figure S1, respectively. Thus, those TSs were confirmed to be in the reaction channel.

**Figure 2:**
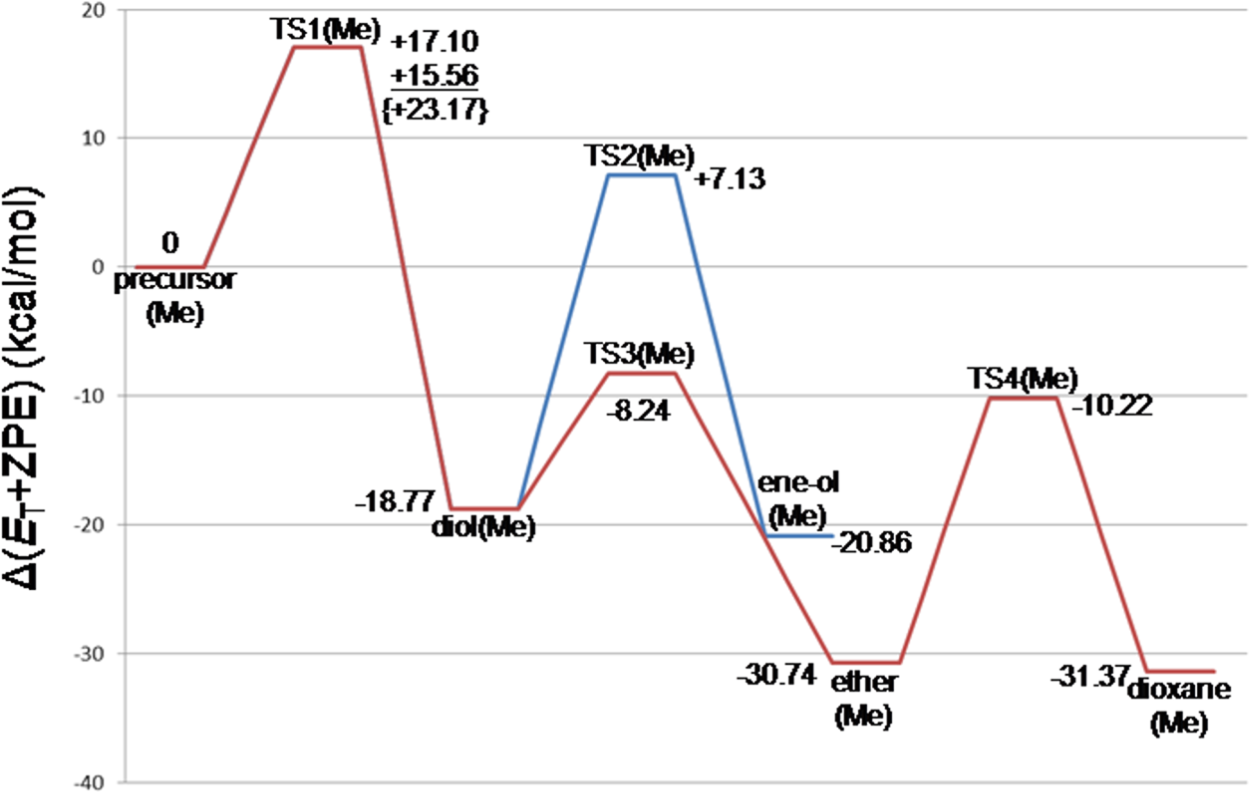
Energy changes (in kcal/mol) of the propene Prins reaction calculated by B3LYP/6-311+G(d,p) SCRF=(PCM, solvent = mwater)// B3LYP/6-31G(d) ZPE. The corresponding geometries are shown in Figure 1 and Figure S1. *E*_T_ stands for the total energy. At TS1(Me), while B3LYP/6-311+G(d,p) SCRF=PCM and M06-2X/6-311+G(d,p) SCRF=PCM energies are similar, the {ωB97XD/6-311+G(d,p) SCRF=PCM} energy seems to be overestimated in spite of the similarity of the three geometries in Figure 1.

Figure 2 exhibits geometry-dependent energy changes for the transition states depicted in Figure 1 and Figure S1. TS1(Me) was found to be the rate-determining step. The butane-1,3-diol, diol(Me), is the first stable intermediate (Δ(*E*_T_ + ZPE) = −18.77 kcal/mol). From diol(Me), two TSs, TS2(Me) and TS3(Me), were obtained. TS3(Me) leading to the hemiacetal intermediate, ether(Me), is much more likely than TS2(Me) leading to the allylic alcohol, ene-ol(Me). In fact, the latter is not formed under the reaction conditions in Scheme 2. The hemiacetal intermediate, ether(Me), is remarkably stable (Δ(*E*_T_ + ZPE) = −30.74 kcal/mol). The stability is almost the same as that of the product, dioxane(Me), (Δ(*E*_T_ + ZPE) = −31.37 kcal/mol). This energetic result suggests that both the ether(Me) and dioxane(Me) are products in the propene Prins reaction, whereas the ether(Me) species has not been reported so far.

### The styrene reaction

**Figure 3:**
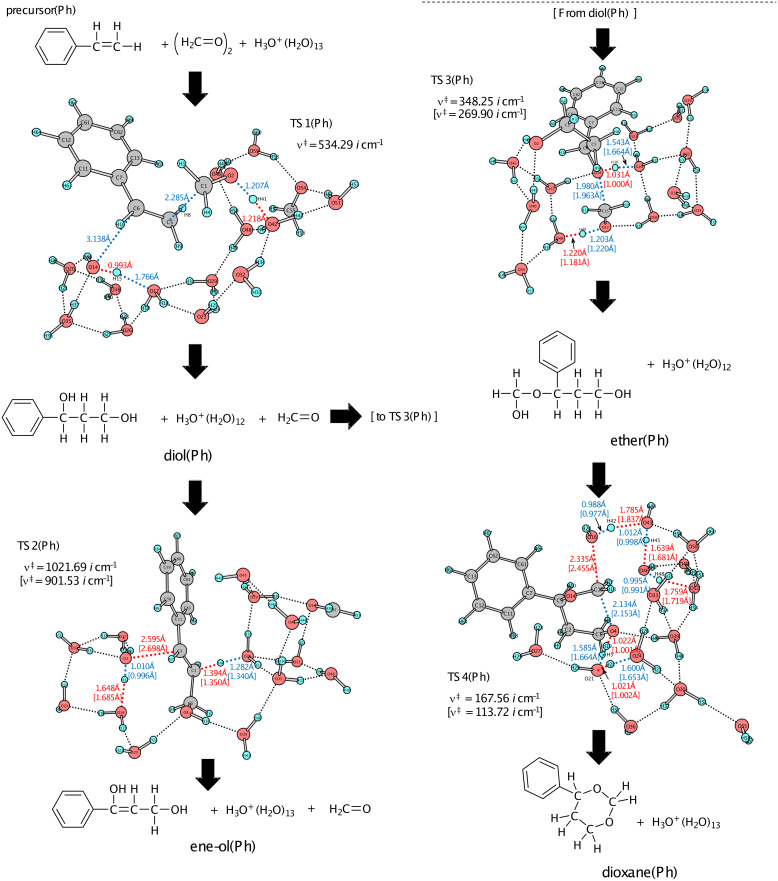
Geometries of the transition states (TSs) of the Prins reaction of styrene + (formaldehyde)_2_ + H_3_O^+^(H_2_O)_13_. Those of intermediates and products are exhibited in Figure S3 of Supporting Information File 1. "(Ph)" denotes the styrene (R–HC=CH_2_, R = phenyl) reaction. The geometry of TS1(Ph) by B3LYP/6-311+G(d,p) is shown in Figure S4 as TS1’(Ph).

Figure 3 shows geometries of four TSs, and Figure S3 shows those of precursor(Ph), diol(Ph), ene-ol(Ph), ether(Ph) and dioxane(Ph). Geometric changes similar to those of the propene Prins reaction were obtained, i.e., precursor(Ph) → TS1(Ph) → diol(Ph) [→ TS2(Ph) → ene-ol(Ph)] → TS3(Ph) → ether(Ph) → TS4(Ph) → dioxane(Ph). Different from the reaction pattern shown in Scheme 5, the cation center is at H_3_O^+^ in the intermediates and product. At TS1(Ph), the incipient C(6)···O(14) bond distance (= 3.138 Å) is extraordinarily larger than the standard one (ca. 1.85 Å) for the C···O-forming TS. For instance, it was calculated to be 1.833 {1.879} Å in the first TS of the acid-catalyzed hydrolysis of ethyl acetate by B3LYP/6-31G(d) {M062X/6-311G(d,p)} in our recent work [40]. Through the B3LYP/6-311+G(d,p) geometry optimization, TS1'(Ph) was obtained as the carbocation (X) formation TS shown in Figure S4. Here, the C(6)···O(14) formation is not involved and the concerted diol(Ph)-formation TS could not be obtained. The character of TS1(Ph) needs to be investigated in more detail and will be discussed in the next subsection.

**Figure 4:**
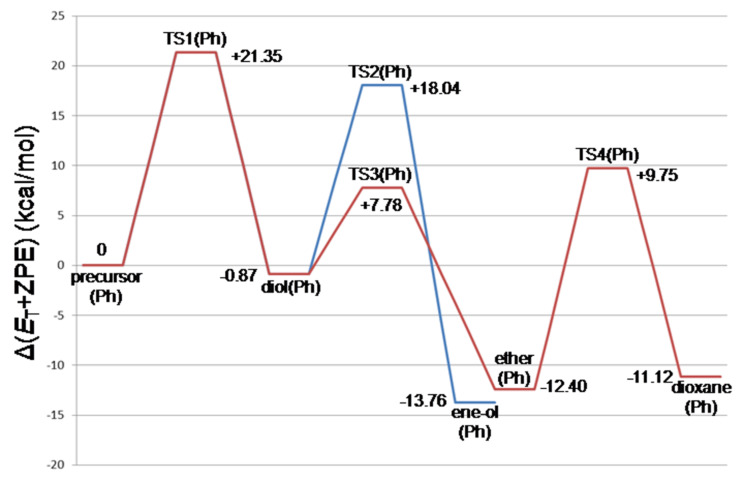
Energy changes (in kcal/mol) of the styrene Prins reaction calculated by B3LYP/6-311+G(d,p) SCRF = (PCM, solvent = water)// B3LYP/6-31G(d) ZPE. The corresponding geometries are shown in Figure 3 and Figure S3.

Figure 4 shows the energy changes of the styrene Prins reaction. They are compared with those of the propene Prins reaction (Figure 2). The rate determining step is again TS1(Ph). A noticeable difference is found in the contrast of the stability order, ene-ol(Ph) > ether(Ph) versus ene-ol(Me) < ether(Me). The 3-phenyl-2-propenol (cinnamyl alcohol) is an allylic alcohol with the π conjugation of the phenyl ring and is thought to be the source of the stability of ene-ol(Ph). However, TS2(Ph) has a large activation energy, +18.04 kcal/mol. Thus, while ene-ol(Ph) is thermodynamically favorable, it is unfavorable kinetically. The energy of ether(Ph), −12.40 kcal/mol, is again similar to that of dioxane(Ph), −11.12 kcal/mol. Both ether(Ph) and dioxane(Ph) may be regarded as products of the styrene Prins reaction. In this respect, the equilibrium depicted in Scheme 3 is not for the (dioxane–diol) pair but for the (dioxane–ether) pair. The product 1,3-dioxane may be obtained with aid of the hygroscopy of the 45−55% sulfuric acid. The water is taken off by H_2_SO_4_, and according to Le Chatelier's principle the equilibrium is shifted toward the dioxane side. Formation of the 1,3-diol in Scheme 3 would arise not from the equilibration with the dioxane but from the high-temperature reflux conditions for the endothermic step, i.e., ether → 1,3-diol.

### The carbocation intermediate (X)

In the Prins reaction of styrene, it is critical whether the first transition state leads to diol(Ph) or to the carbocation X(Ph). The intervention of X(Ph) was examined by an extended model shown in Figure S5. In the model, the initial geometry for the optimization was made of that of X(Ph) in Figure S4 and seven additional water molecules (atom numbers, from 68 to 88). Through the optimization by B3LYP/6-31G(d) and B3LYP/6-311+G(d,p), the initial carbocation was found to be converted to the 1,3-diol as shown in the lower side of Figure S5. Thus, the carbocation would intervene when the size of the water cluster surrounding the reactants (alkene and formaldehyde) is small. While this condition corresponds to the reaction in a binary solvent such as acetone–water, the reaction in aqueous media would not involve the carbocation X.

**Figure 5:**
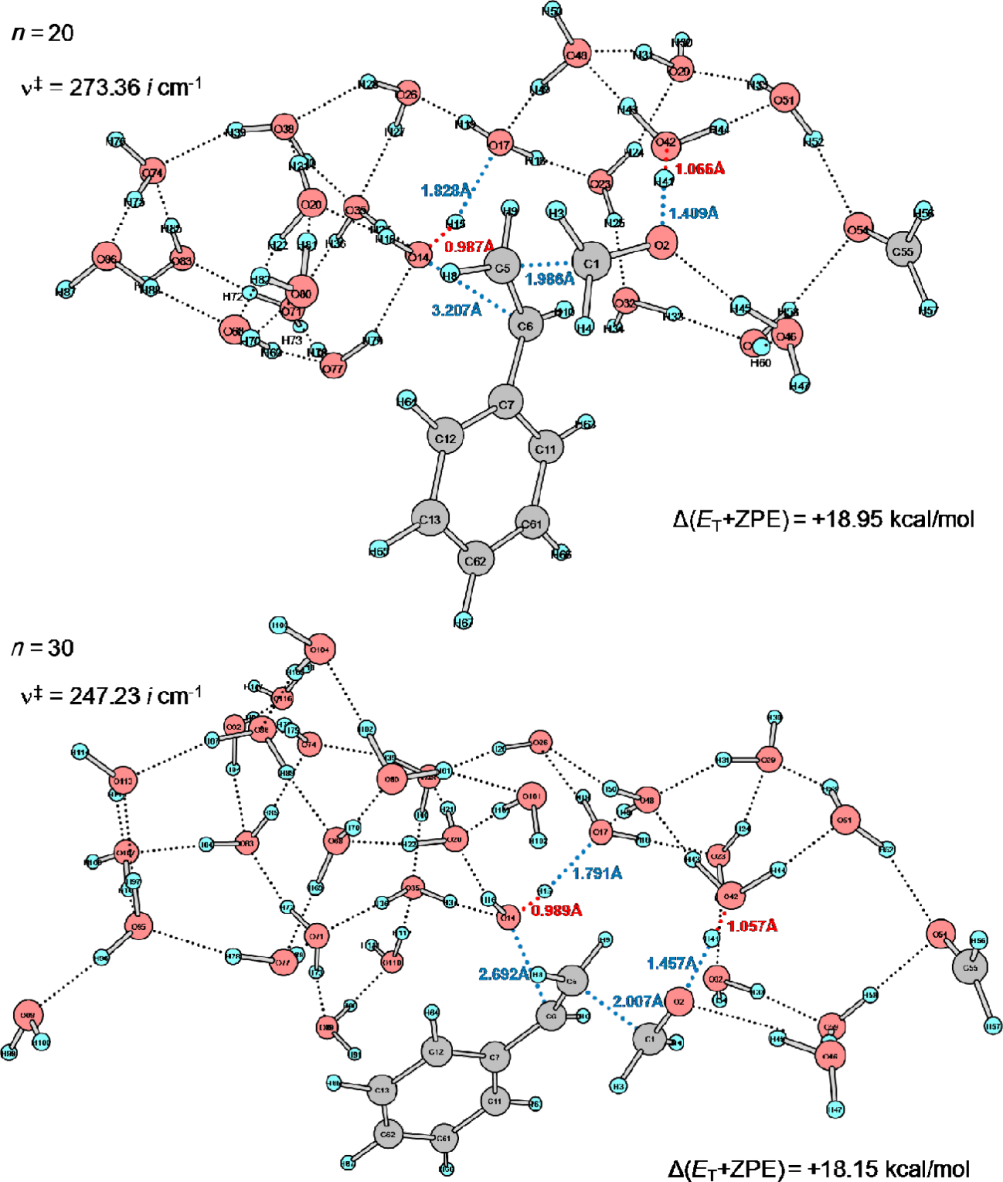
TS1(Ph) geometries of *n* = 20 and *n* = 30 in the reacting system of styrene + H_3_O^+^(H_2_O)*_n_* + (H_2_C=O)_2_ calculated by B3LYP/6-31G(d).

Dependence of the number of water molecules on the geometries of TS1(Ph) was investigated by the use of two extended models, styrene + H_3_O^+^(H_2_O)*_n_* + (H_2_C=O)_2_ (*n* = 20 and 30). The *n* = 13 TS1(Ph) is shown in Figure 3. The *n* = 20 and 30 TS1(Ph) geometries are shown in Figure 5.

While the central part of the *n* = 20 geometry is similar to that of the *n* = 13 one, the incipient O(14)···C(6) bond in the *n* = 30 TS1(Ph) is shorter (2.692 Å) than those of the *n* = 13 and *n* = 20 TSs. This result indicates that the extended model of the *n* = 30 TS1(Ph) expresses clearly the 1,3-diol formation.

## Conclusion

**Scheme 7:**
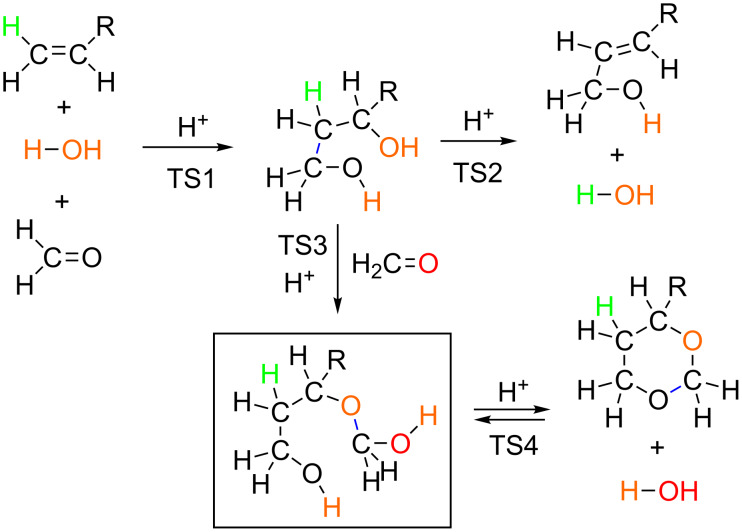
Summary of the present calculated results. The ether in the box is the new intermediate found in this work. The blue line stands for the newly formed covalent bond at each step.

In this work, two Prins reactions were investigated by the use of B3LYP calculations. A model composed of R–CH=CH_2_ + H_3_O^+^(H_2_O)_13_ + (H_2_C=O)_2_, R = Me and Ph, was employed to trace reaction paths. The result is summarized in Scheme 7.

The 1,3-diol is formed concertedly in the rate-determining step, TS1. From the diol, the ene-ol is afforded in the E2 (bimolecular elimination, TS2) pathway. Most likely, the addition (TS3) of the second formaldehyde to the 1,3-diol leads to the new intermediate (ether or hemiacetal). Ring closure from the ether gives the product, 1,3-dioxane. The 1,3-dioxane is in equilibrium with the ether.

It is critical whether TS1 goes to the 1,3-diol or to the carbocation X. While the intervention is suggested to depend on the concentration of water, in aqueous media the cation is unlikely owing to the high nucleophilicity of the large water cluster.

## Supporting Information

File 1 Geometries of the precursor, intermediates and products, and other related geometries.
