# Supplementary material for: A new intermediate in the Prins reaction
Source: Beilstein J Org Chem. 2013 Mar 5;9:476–85. doi: 10.3762/bjoc.9.51 (PMC3596789; doi:10.3762/bjoc.9.51)
Supplement: File 1 — Geometries of the precursor, intermediates and products, and other related geometries. [file Beilstein_J_Org_Chem-09-476-s001.pdf]

Supporting Information
for

A new intermediate in the Prins reaction

Shinichi Yamabe,[1]* Takeshi Fukuda[2] and Shoko Yamazaki[2]

Address: [1]Fukui Institute for Fundamental Chemistry, Kyoto University

Takano-Nishihiraki-cho 34-4, Sakyou-ku, Kyoto 606-8103, Japan and [2]Department of

Chemistry, Nara University of Education, Takabatake-cho, Nara 630-8528, Japan

Email: Shinichi Yamabe - yamabes@fukui.kyoto-u.ac.jp

*Corresponding author

Geometries of the precursor, intermediates and products, and other related geometries.

Figure S1: Geometries of intermediates and products of the propene Prins reaction. Page S2

Figure S2: Geometries at the 200 femtoseconds passed from TS1(Me), TS2(Me), TS3(Me), TS4(Me) in Figure 1 and TS1(Ph) in Figure 3. Pages S3–S7

Figure S3: Geometries of the precursor, intermediates and products of the styrene Prins reaction. Pages S8–S9

Figure S4: A carbocation [X(Ph)] formation path calculated by B3LYP/6-311+G(d,p). Page S10

Figure S5: The initial geometry composed of that of the carbocation X(Ph) and $(H_2O)_7$ is converted into that of diol(Ph) and $(H_2O)_7$ both by B3LYP/6-31G(d) and by B3LYP/6-311+G(d,p). The molecular formula of the system is $C_{10}H_{55}O_{23}^+$. Pages S11–S12

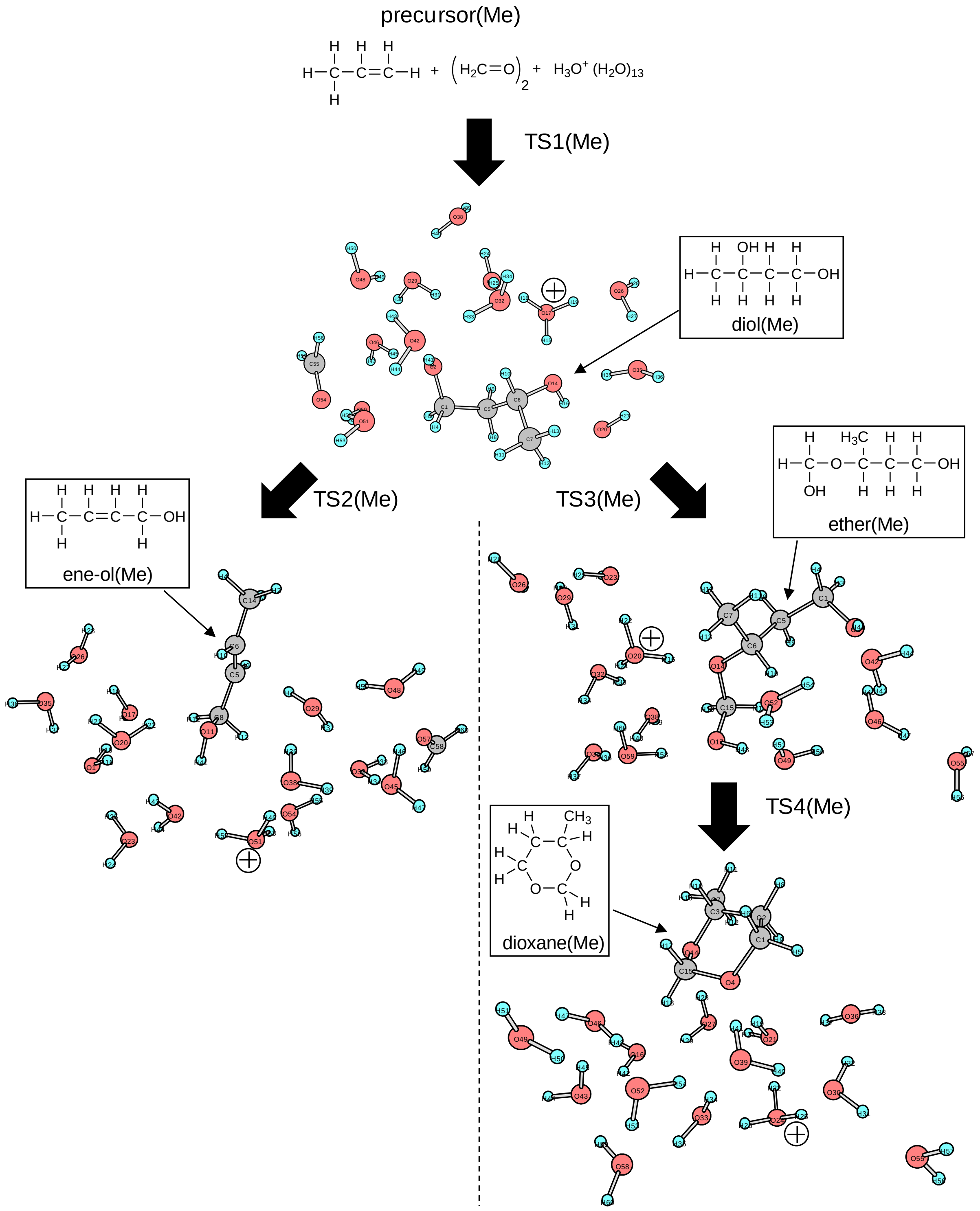

Figure S1: Geometries of intermediates and products of the propene Prins reaction. TS geometries are shown in Figure 1.

TS1(Me)

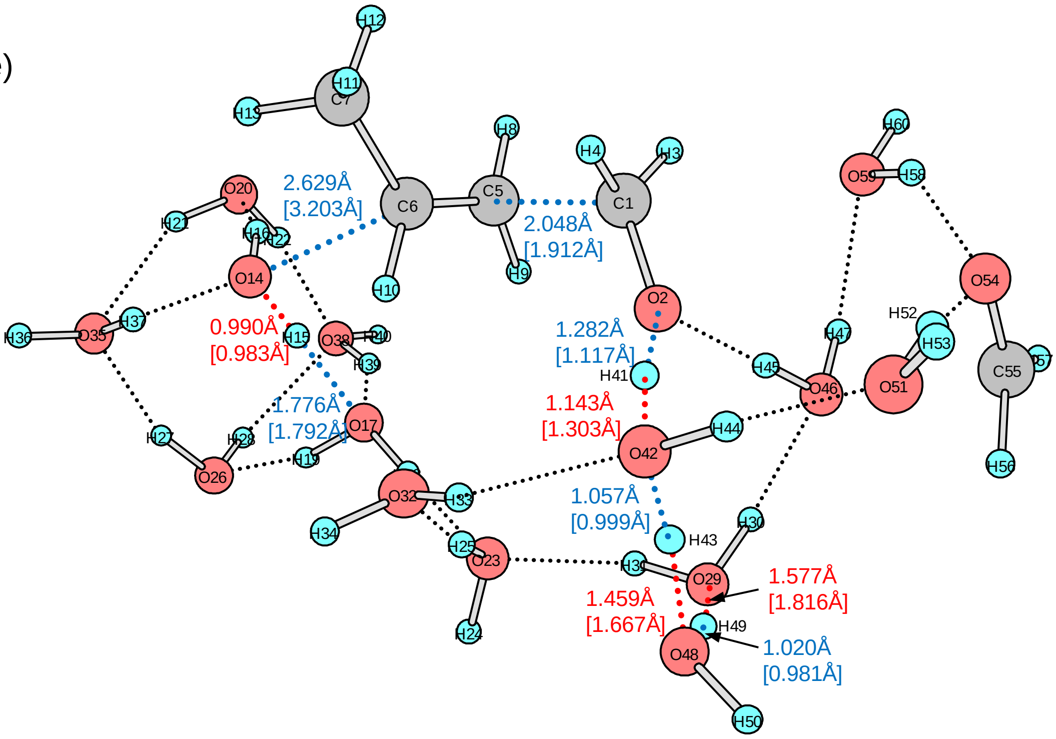

200 femtoseconds

ADMP-1(Me)

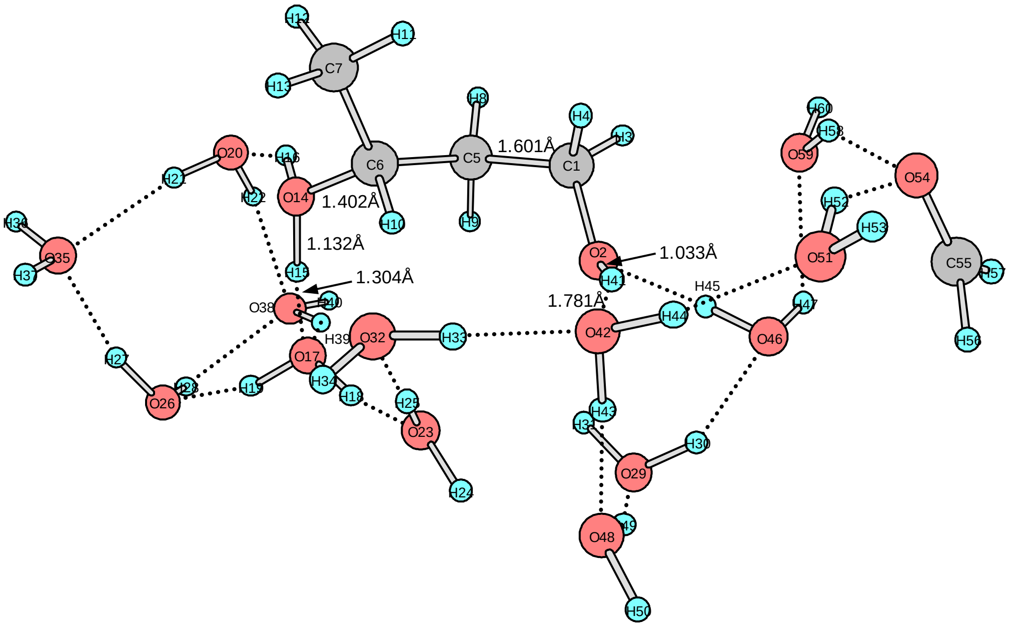

TS2(Me)

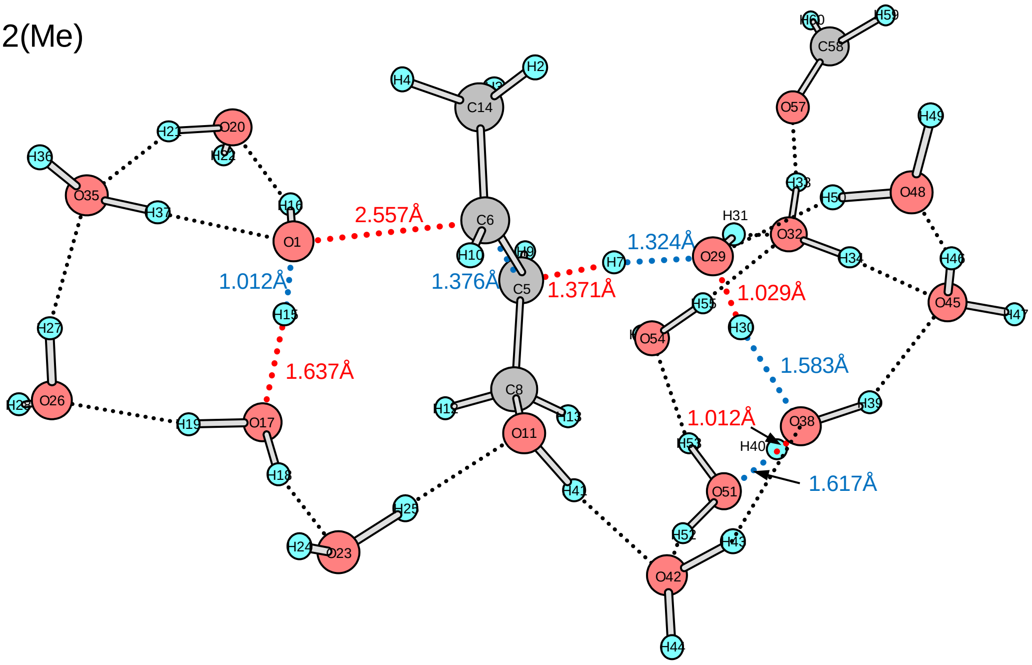

200 femtoseconds

ADMP-2(Me)

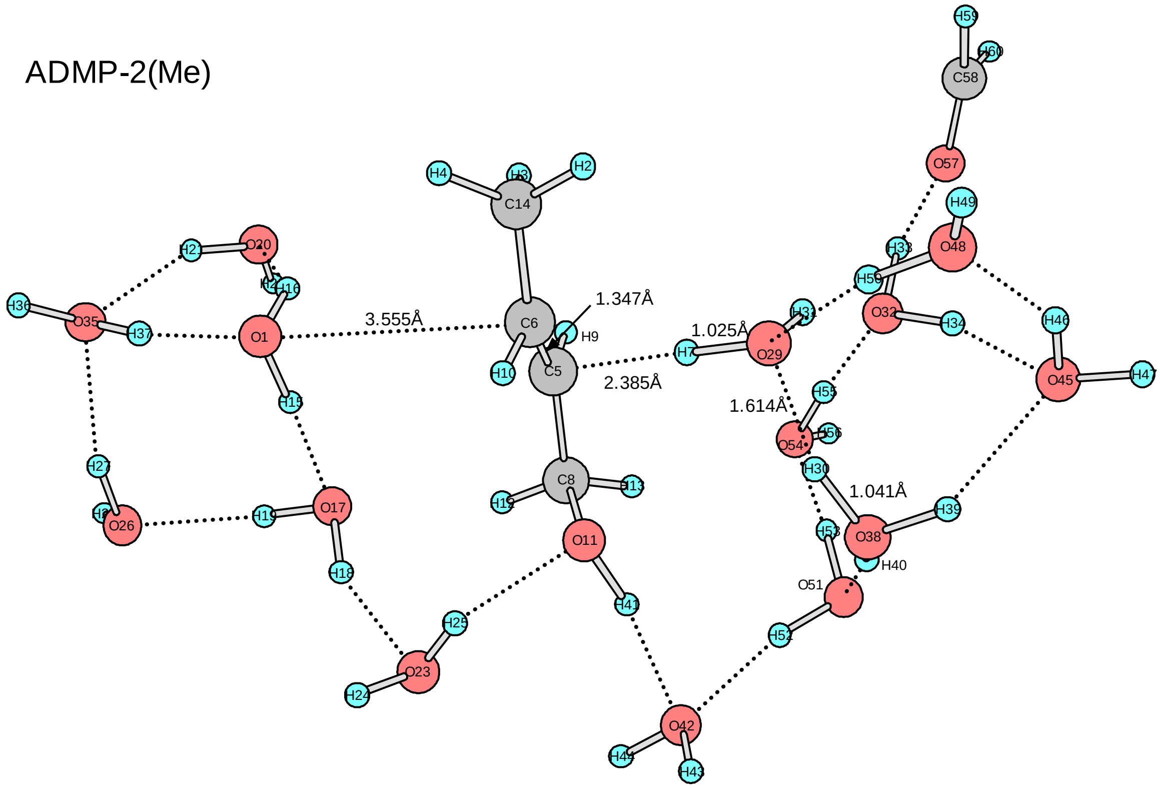

TS3(Me)

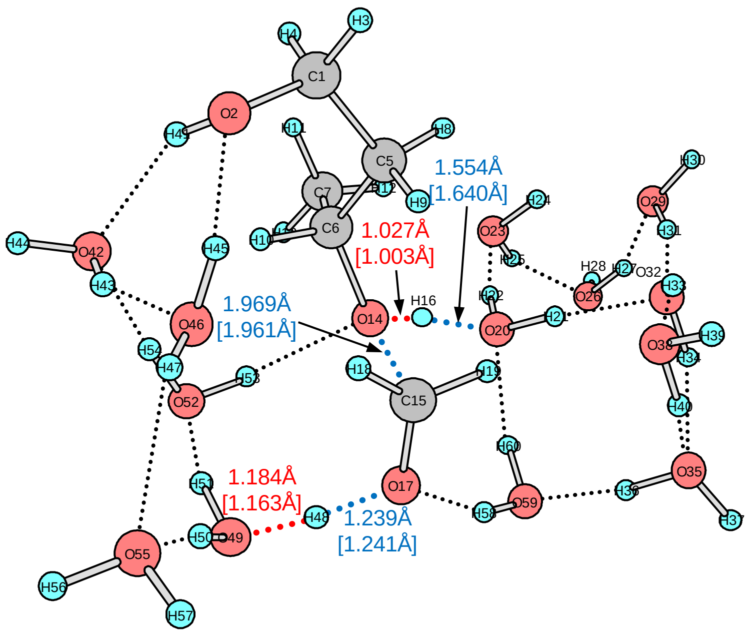

200 femtoseconds

ADMP-3(Me)

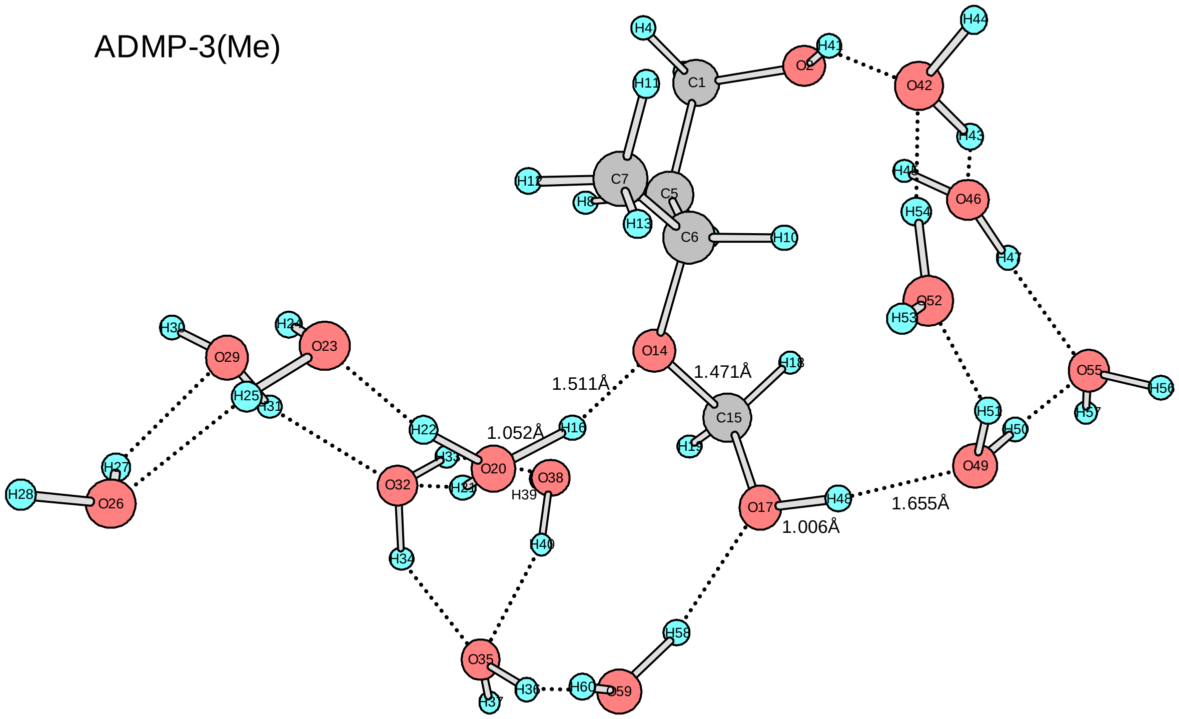

TS4(Me)

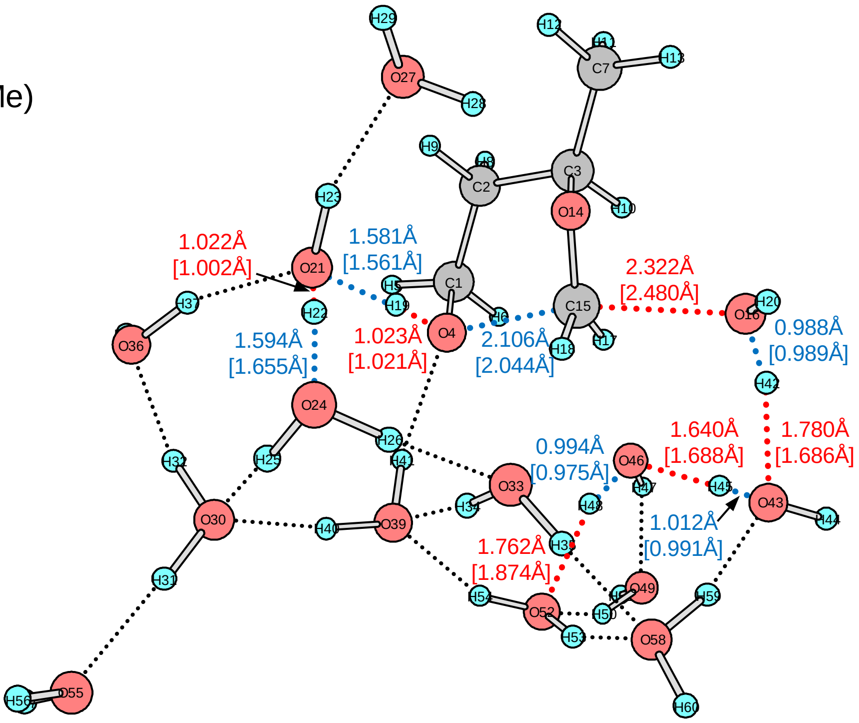

200 femtoseconds

ADMP-4(Me)

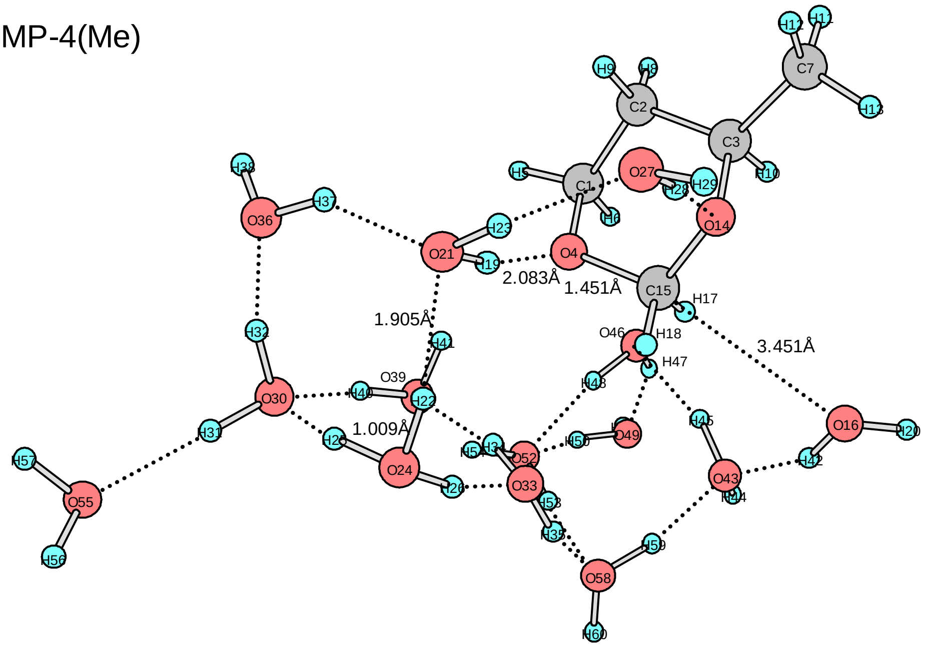

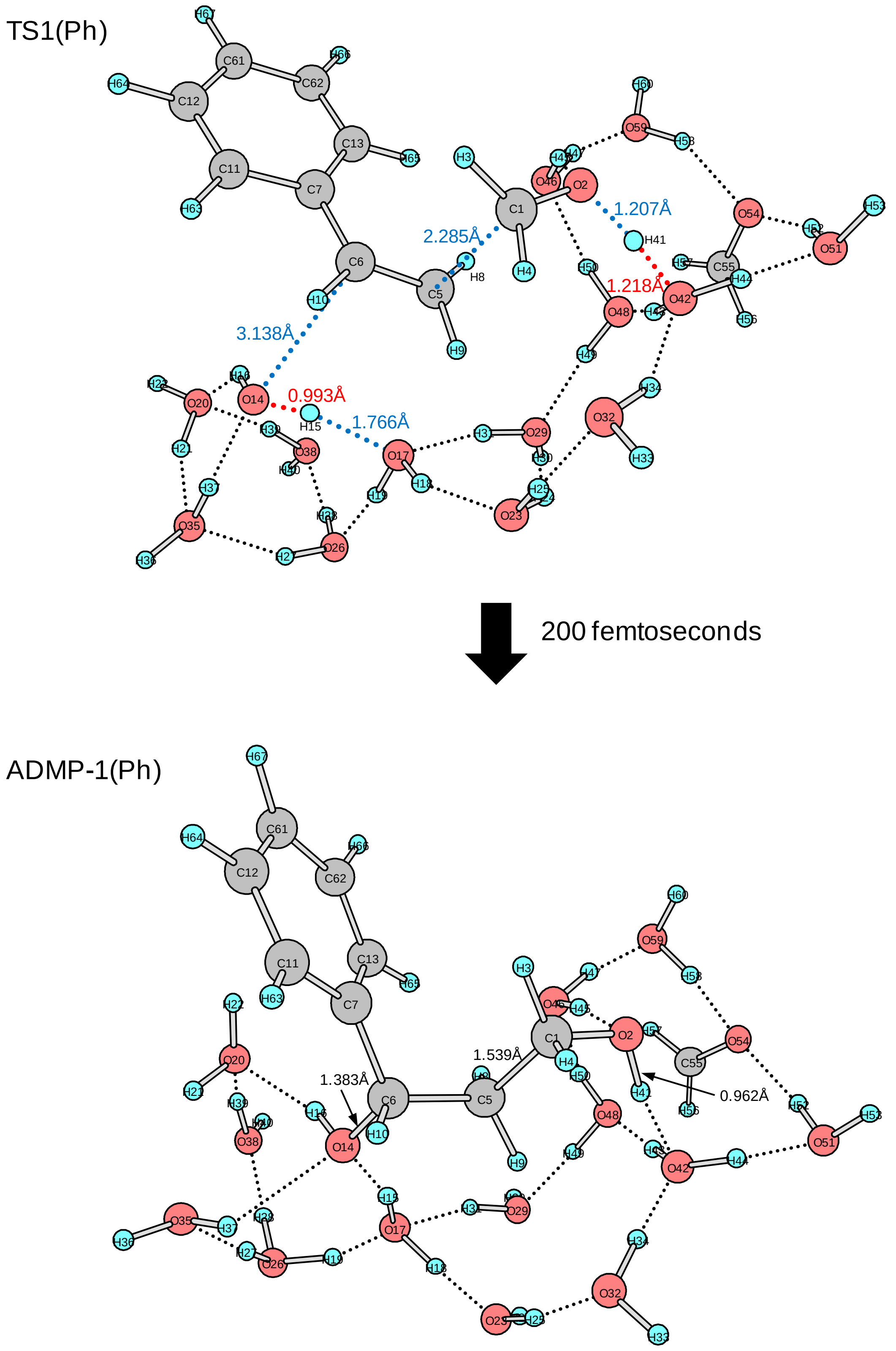

Figure S2: Geometries at the 200 femtoseconds passed from TS1(Me), TS2(Me), TS3(Me), TS4(Me) in Figure 1 and TS1(Ph) in Figure 3.

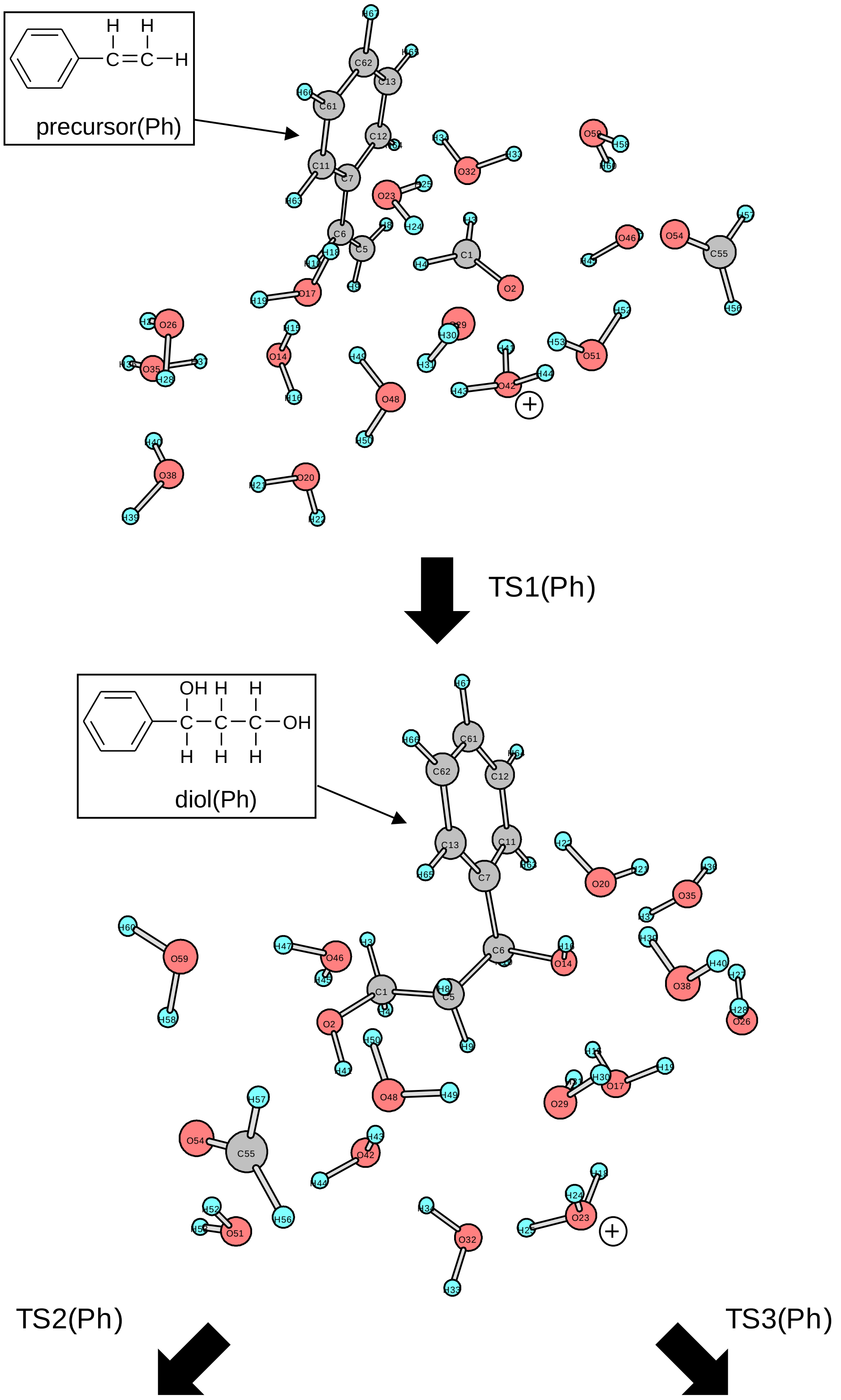
precursor(Ph)
TS1(Ph)
diol(Ph)
TS2(Ph)
TS3(Ph)

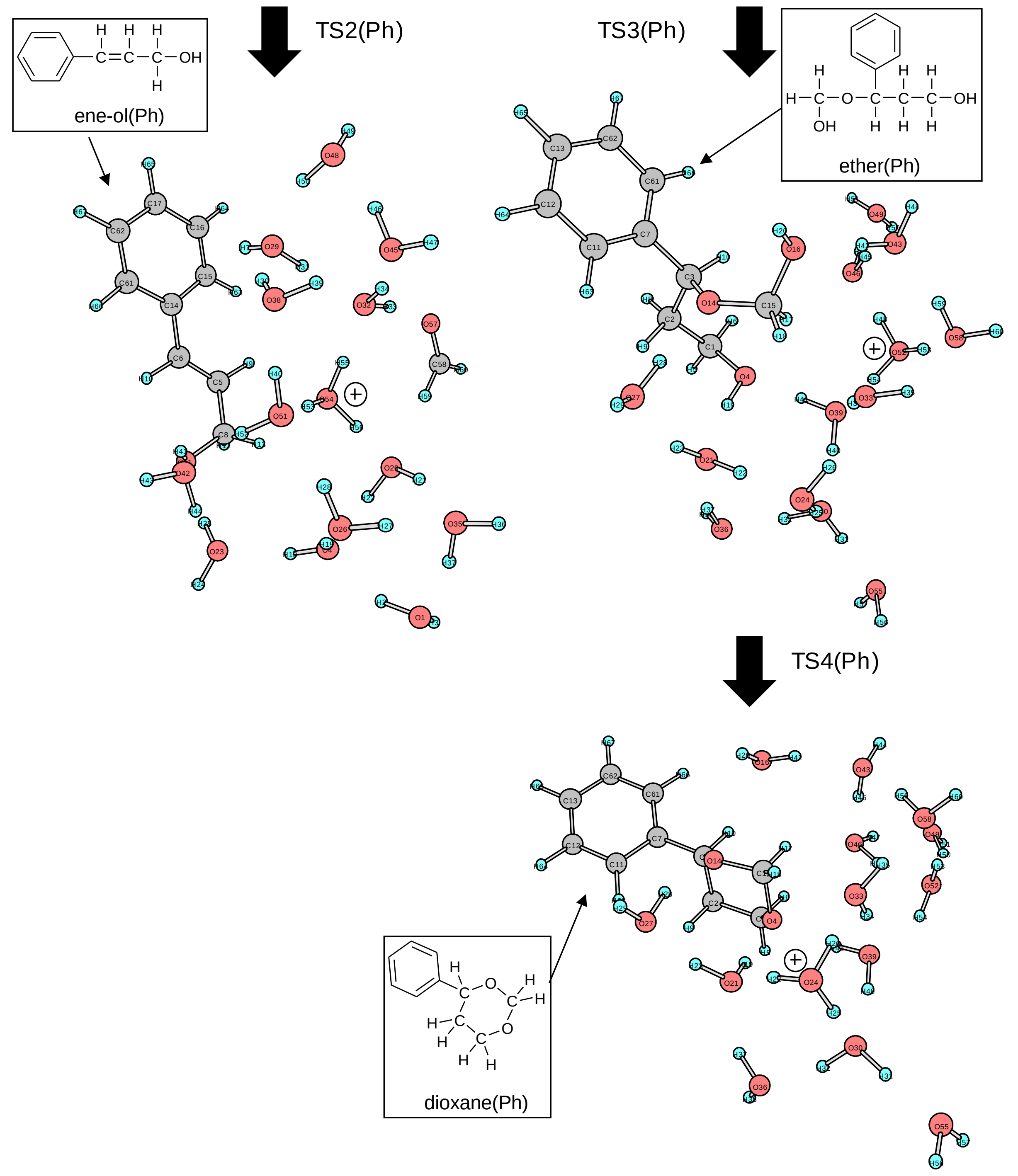

Figure S3: Geometries of the precursor, intermediates and products of the styrene Prins reaction. TS geometries are shown in Figure 3.

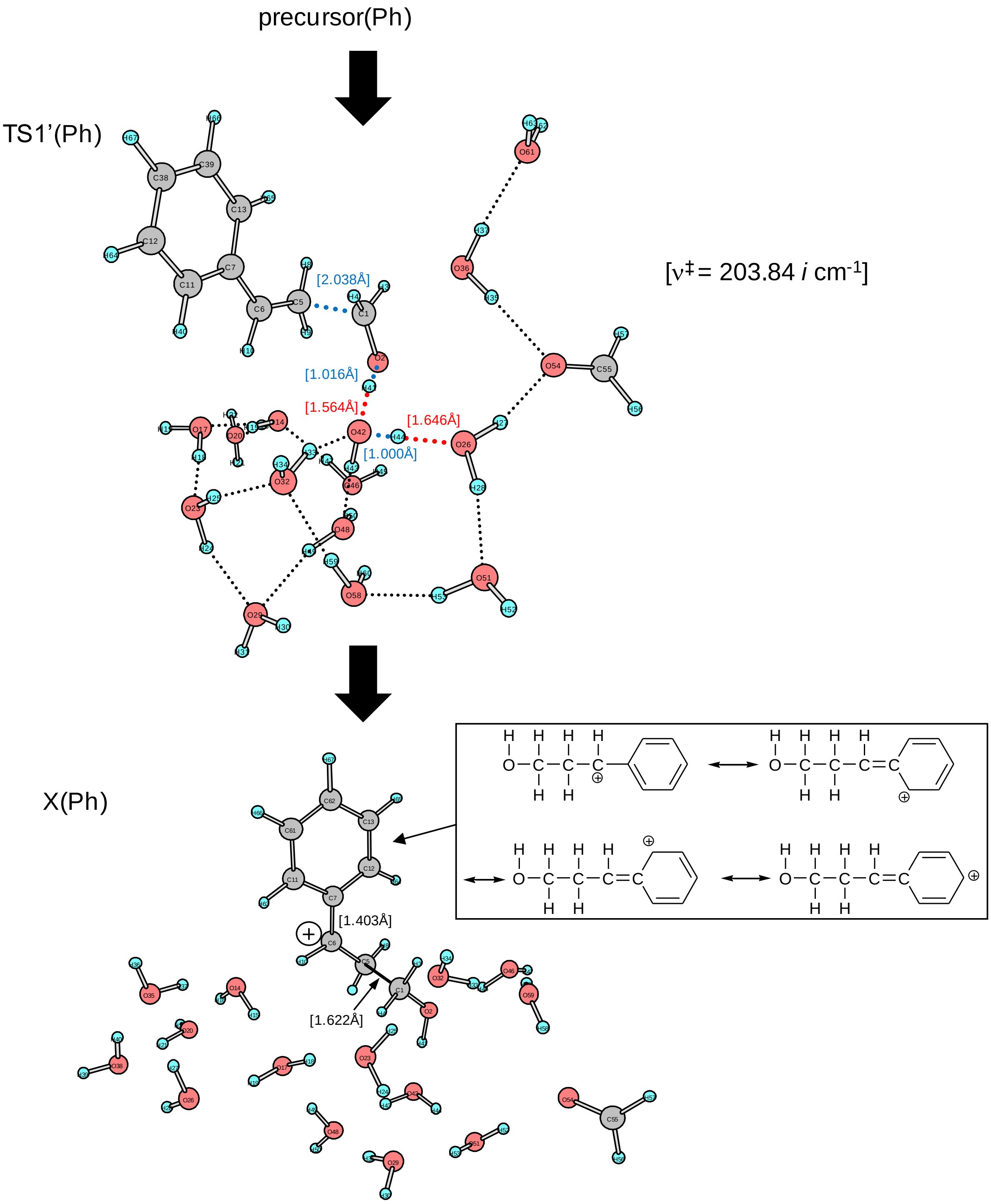

Figure S4: A carbocation [X(Ph)] formation path calculated by B3LYP/6-311+G(d,p). The geometry of precursor(Ph) is shown in Figure S3. The cation X(Ph) is stabilized by canonical resonance structures exhibited in the box.

The initial geometry

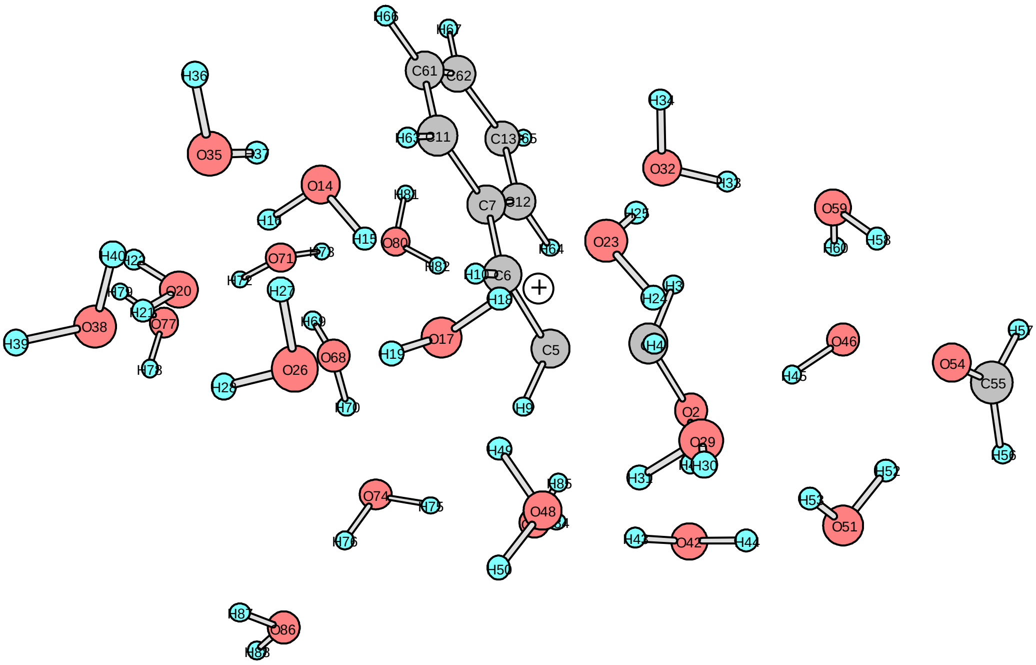

B3LYP/6-31G(d)

The optimized geometry

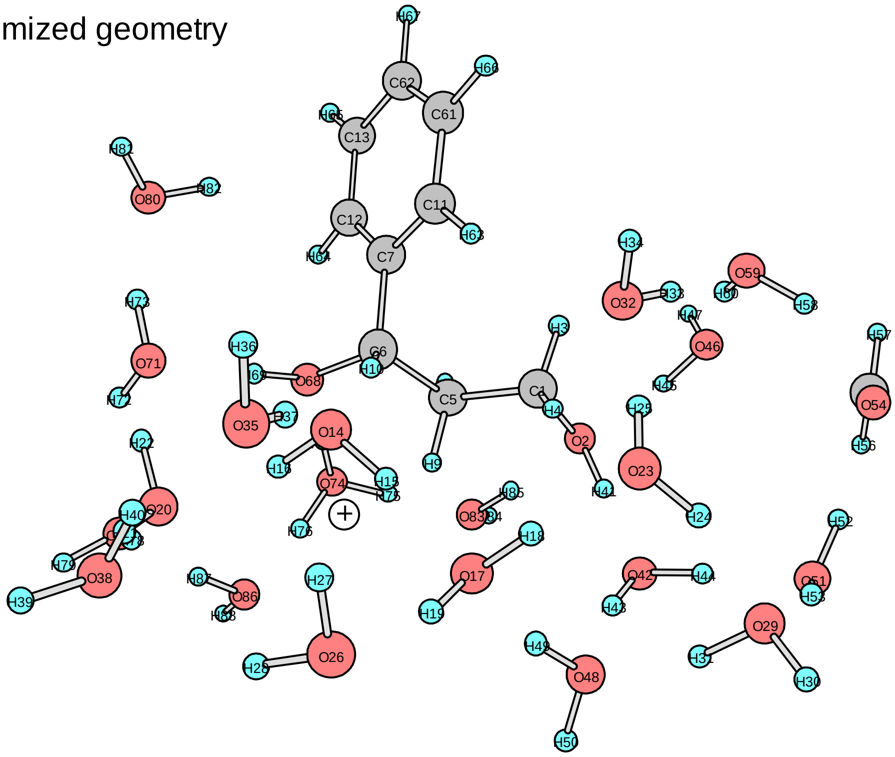

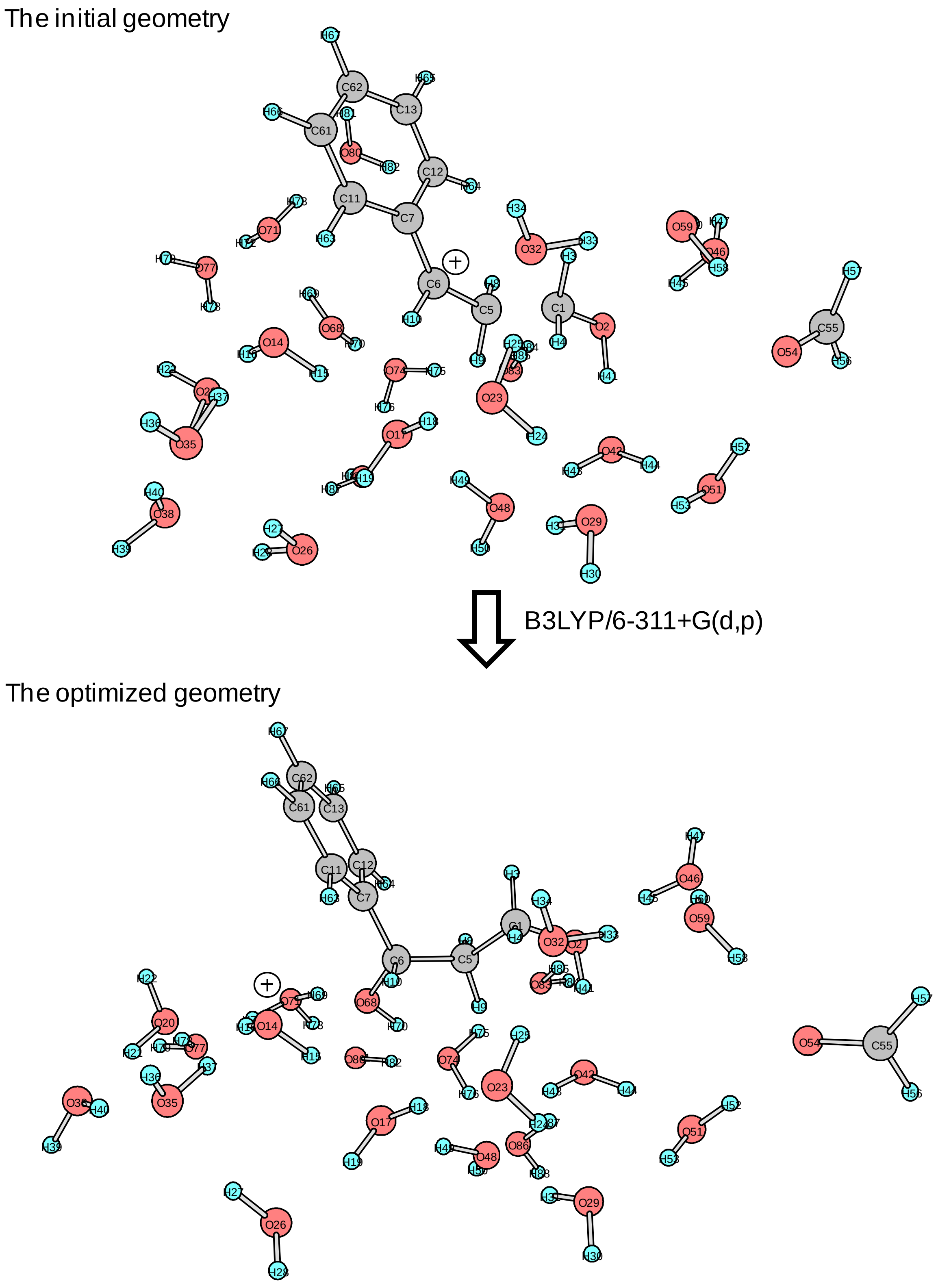

Figure S5: The initial geometry composed of that of the carbocation X(Ph) and $(H_2O)_7$ is converted into that of diol(Ph) and $(H_2O)_7$ both by B3LYP/6-31G(d) and by B3LYP/6-311+G(d,p). The molecular formula of the system is $C_{10}H_{55}O_{23}^{+}$.
